# Privacy Protection in Using Artificial Intelligence for Healthcare: Chinese Regulation in Comparative Perspective

**DOI:** 10.3390/healthcare10101878

**Published:** 2022-09-27

**Authors:** Chao Wang, Jieyu Zhang, Nicholas Lassi, Xiaohan Zhang

**Affiliations:** 1 Guanghua Law School, Zhejiang University, Hangzhou 310008, China; 2 School of Law, Zhejiang University City College, Hangzhou 310015, China

**Keywords:** artificial intelligence, big data, data collection, healthcare information, patient privacy, personal information, privacy protection

## Abstract

Advanced artificial intelligence (AI) technologies are now widely employed in China’s medical and healthcare fields. Enormous amounts of personal data are collected from various sources and inserted into AI algorithms for medical purposes, producing challenges to patient’s privacy. This is a comparative study of Chinese, United States, and European Union operational rules for healthcare data that is collected and then used in AI functions, particularly focusing on legal differences and deficiencies. The conceptual boundaries of privacy and personal information, the influence of technological development on the informed consent model, and conflicts between freedom and security in rules of cross-border data flow were found to be key issues requiring consideration when regulating healthcare data used for AI purposes. Furthermore, the results indicate that the appropriate balance between privacy protections and technological development, between individual and group interests, and between corporate profits and the public interest should be identified and observed. In terms of specific rule-making, it was found that China should establish special regulations protecting healthcare information, provide clear definitions and classification schemas for different types of healthcare information, and enact stricter accountability mechanisms. Examining and contrasting operational rules for AI in health care promotes informed privacy governance and improved privacy legislation.

## 1. Introduction

According to the World Health Organization (WHO), artificial intelligence (AI) has “enormous potential for strengthening the delivery of healthcare and medicine” [1]. AI technologies are currently being used for various medical purposes, including disease detection and diagnosis, medical imaging, personalized disease treatment, and drug development [2]. In epidemiological and public health related fields, AI can assist in deploying interventions such as disease surveillance, outbreak response, and the management of healthcare systems [3]. At the same time, AI applications bring risks and challenges to human rights and medical ethics. For instance, AI may threaten personal privacy, affect human decision-making autonomy and human dignity, and generate algorithmic discrimination, among others. 

One outstanding issue in recent years concerns the protection of patient privacy rights when AI is deployed for healthcare purposes. When patient privacy is unprotected, negative consequences, such as employment discrimination and increased long-term healthcare costs, are more likely to occur [4]. Historically, healthcare information was recorded and stored on physical documents. Protecting privacy largely involved patient confidentiality among medical staff within medical institutions. Nowadays, patient information is increasingly recorded digitally and electronically, where it is often sent to a larger collection of relatively fluid healthcare information and used for numerous purposes. Since AI related medical technology is based on the collection and use of patient information, privacy protection issues are becoming increasingly complex in an era when information sharing has never been so convenient and profitable. 

Chinese AI technology in healthcare has advanced rapidly in recent years. AI technology is now applied to critical medical scenarios such as medical imaging, assisted decision-making, medical robots, and drug research and development [5]. Since 2020, several products developed by China’s medical AI industry have been approved by the National Medical Products Administration (NMPA) under Class III classification. This includes products benefiting orthopedics, ophthalmology, and the cardiovascular and respiratory systems [6]. Market sectors developing and employing cutting-edge health care technologies, such as AI-related drug research and medical imaging, have maintained high growth rates. China’s medical AI market is expected to exceed 4.5 billion USD in 2025 [7]. The application of AI in healthcare is an important strategic direction for the Chinese government. For example, the “Healthy China 2030” planning outline states that China will actively promote a deep integration of big data technology with healthcare services, in which AI-centered medical care is a key driving force [8]. China’s State Council, Ministry of Industry and Information Technology (MIIT), National Medical Products Administration (NMPA), and other ministries have adopted a series of policies promoting AI-related medical imaging, smart hospitals, medical robots, and other related sub-directions. 

However, privacy rights have historically been underrated in China. Privacy laws and regulations are generally fragmented, vague, and poorly enforced. There are frequent incidents of personal information infringement, and breaches in medical data have eroded the public’s confidence in data processing [9,10]. These incidents, along with a sociopolitical environment where privacy rights are generally subservient to other social goals, create obstacles to the sustainable development of medical AI in China.

China’s current legislation on privacy protection in medical AI is largely based on the Personal Information Protection Law (PIPL) and the Civil Code, as well as relevant national standards. This includes specific rules pertaining to individual consent and authorization for data processing, wherein subjects receive, at a minimum, the purpose for the information processing, along with its manner and scope. However, there are no specific laws and regulations enforced for big data processing and use (e.g., algorithmically generated probabilities and predictions) in the Chinese healthcare industry [11].

Academic discussions of patient privacy protection in healthcare are ongoing. Studies have explored how group dimensions influence protection mechanisms for privacy interests [12], the possible costs of data restrictions [13], and defects in the emerging consensus on “ethical AI” from the perspective of human rights regulations [14]. However, research focusing on regulatory issues in the Chinese context is rare, and certain issues remain unclear.

This study examines the status of Chinese privacy protection legislation and locates deficiencies when compared to the more mature privacy protection models instituted in the U.S. and EU. The current Chinese privacy governance framework is detailed, regulatory weaknesses are located, and pathways for improvement are formed. Recommendations, resulting from this examination, for how Chinese law can be improved are put forward.

The structure of this article is as follows: Section 2 introduces the sources of data and the research methodology of this study. Section 3 empirically examines current Chinese patient privacy legislation, along with the corresponding judicial practices, and it compares Chinese regulations with those of the U.S. and EU. Section 4 discusses the dilemmas produced by medical AI in privacy protection, including the conceptual boundaries between personal information and personal privacy, and the challenging nature of non-identifiable personal information as it exists within the consent authorization model. Section 5 concludes the article with recommendations, including balancing values at the macro level and how specific systems and rules require improvements at the micro level.

## 2. Materials and Methods

### 2.1. Research Data

An empirical investigation into current developments in Chinese medical AI privacy protections was conducted. To this end, corresponding reports issued by major research institutions were studied, including the 36 Krypton Institute, Eggshell Research Institute, and iResearch. Concerning the protection and infringement of healthcare information, official reports from China’s National Computer Network Emergency Technology Handling Coordination Center (CNCERT) were analyzed, such as its annual “China Internet Network Security Report”. News reports on personal information leaks were also included in the examination. For cases decided by judicial organs, information from China Judgments Online, which is a repository of data and information of the Supreme People’s Court, was collected and examined. Regarding information on privacy protection policies in China, the official websites of corresponding Chinese authorities were reviewed, including the Ministry of Public Security and the State Internet Information Office of China (CAC). For detailed information on Chinese, U.S., and EU laws and regulations, professional legal databases such as Chinalawinfo, HeinOnline, Westlaw, and LexisNexis were exercised using the keywords “privacy”, “personal information”, “informed consent”, and “cross-border data flow”, among others.

### 2.2. Research Methods

A comprehensive literature review of Chinese developments in privacy protection law for healthcare data used in AI operations was conducted. An empirical examination of Chinese privacy protection laws and regulations, law enforcement practices, and judicial applications provide the foundation for the study. Also, an investigation into actions taken by various regulatory authorities in personal information protection cases was conducted. 

**Figure 1:**
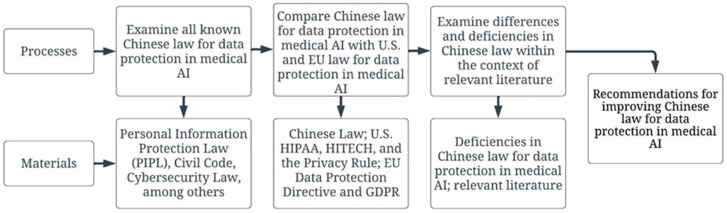
The processes and materials employed in the examination.

Additionally, Chinese, US and EU patient privacy protection laws were compared. All relevant patient privacy protection laws from China, the U.S. and EU were collected from formal and current government sources, placed within an extensive rubric for comparison and instructional purposes, and examined for differences and deficiencies. Once differences or deficiencies were located, further research into formal explanations for the existence of these specific laws or lack thereof, from government and other legal sources, was conducted. The capacity to provide recommendations for future legislation is improved when unique laws, legal differences, and deficiencies are thoroughly detailed and understood. Comparing different legal provisions on privacy protection highlighted deficiencies in existing privacy protection law in China. These comparisons produced recommendations for reforming Chinese law (see Figure 1).

## 3. Results

### 3.1. Origin, Regulatory Status, and Judicial Practice

The notion that human intelligence could be simulated by advanced computer systems was first proposed by Alan Turing in 1950 with the publication of “Computing Machinery and Intelligence”, which explored machine intelligence and introduced concepts such as the “Turing Test” [15]. The term “artificial intelligence” originated in 1955, when it was used in a research proposal prepared by scientists at Dartmouth College [16], where it was proposed that “every aspect of learning or any other feature of intelligence can in principle be so precisely described that a machine can be made to simulate it”. The digitizing of medical data, fundamental for medical AI growth, originated in the 1960s with the development of the Medical Literature Analysis and Retrieval System (MEDLARS) and other search engines produced by the National Library of Medicine (NLM) [17]. More advanced applications for AI in medical care came into focus in the early to mid-1970s, with the invention of a backward chaining expert system using goal directed control strategies called MYCIN [18]. This system employed AI to detect infection causing bacteria and diagnose blood clotting diseases, though it was never applied in the medical field due to legal and ethical concerns involving computers in medicine [19]. MYCIN technology was influential in spawning the more advanced rule-based system EMYCIN and the massive medical knowledge repository and diagnosis tool INTERNIST (which later became the widely used Quick Medical Reference (QMR) system) [17]. In 2007, IMB unveiled Watson, an open-domain question-answering system using AI technology called DeepQA. Watson’s medical technology, now referred to as Watson Health or Merative, analyzes large quantities of medical information to provide personalized and evidence-based medical recommendations and other clinical decision-making services, operating with a significant focus on cancer treatments [20]. Several IBM contemporaries, such as Google’s DeepMind and Microsoft’s Bio Model Analyzer software, have also played significant roles in the development and use of AI in the medical field during the 21st century [21]. 

Since the beginning of the 21st century, Chinese AI has made significant progress in the medical field. The market size of AI+ core medical software services exceeded two billion USD in 2019, with a year-on-year growth rate of 93.9 percent [22]. The Clinical Decision Support System (CDSS) accounted for the largest portion of this growth, reaching 55.2 percent. At the same time, this rapid development in medical technology has led to an increased risk for breaches in healthcare data. According to the “China Internet Network Security Report 2020”, released by the National Computer Network Emergency Response Technical Team/Coordination Center of China (CNCERT/CC), medical imaging data was exported through domestic networks more than 4.97 million times in 2020, involving 3347 domestic IP addresses [23]. Medical imaging files contain large amounts of private patient information without desensitization. Nearly 400,000 non-desensitized Chinese medical imaging files were exported domestically in the year 2020, accounting for 7.9 percent of the total number of exports [23]. Breaches in personal information may lead to violations of citizens’ privacy, therefore it is urgent and necessary to develop fair and functional personal information security and protection.

In China, the supervision of personal information protection is comprehensively coordinated by the Cyberspace Administration of China (CAC), which directs the Public Security Department, Industry and Information Department, Market Supervision and Management Department, and other related industry authorities exercising supervision and management responsibilities. The CAC and the China Cyberspace Security Association have implemented an application platform (APP) for smartphones and computers to accept complaints and reports on the collection and use of personal information, particularly those relating to law and regulation violations. Since 2021, the platform has received more than 20,000 complaints and reports on personal information protection violations through communicatory channels such as the social media and communications APP WeChat, public telephone numbers, and emails [24]. In 2021, China’s public security authorities launched the “Clean Network 2021” special operation to address the issue of personal information protection. From this initiative, more than 9800 cases of personal information infringement were solved [25].

In terms of judicial practices, China’s Supreme People’s Court noted in its annual work report at the 13th National People’s Congress that Chinese courts have concluded 4098 cases related to various crimes against citizens personal information in 2021, with a year-on-year increase of 60.2 percent [26]. These crimes include the theft and sale of personal ID cards, address books, courier lists, WeChat accounts, and patient information. China Judgments Online contains 1800 cases of privacy disputes, including 46 judgments directly related to privacy disputes with medical institutions [27]. These medical cases largely focused on whether medical personnel and medical institutions illegally disclosed medical records, such as case information, without patient consent. 

In 2021, the Hangzhou Intermediate People’s Court concluded a high-profile privacy dispute known as “China’s First Facial Recognition Case” [28]. This case is significant for the legal regulation of personal information and privacy in China, as the verdict emphasized the need for processors of personal information, and it established that medical staff and medical institutions are to engage in the “lawful, justified, and necessary” collection and use of personal information. This case also initiated private interest litigation on personal information protection, which means that in the future, in addition to civil lawsuits filed by individuals, there may also be group lawsuits, representative lawsuits, and public interest lawsuits by consumer organizations in disputes over privacy protection in the medical and healthcare industries. 

Statistical data on the extent of privacy or data abuse in the Chinese healthcare industry is limited within this emerging and somewhat information-controlled field, however, certain cases shed light on the extent of this growing problem. In August 2022, a hacker allegedly attempted to sell the personal and healthcare data of nearly 50 million users of Shanghai’s compulsory COVID health code application [29]. Data samples provided by the hacker to potential buyers include users’ names, identification numbers, phone numbers, and COVID status. The Shanghai medical data leak follows a massive July 2022 personal data breach, where the personal information of around one billion Chinese citizens was leaked from a police database in Shanghai [9]. In October 2020, Sichuan Lianhao Technologies, an online Chinese medical company, purportedly leaked 24 million medical records, including patients and doctors’ names, identification numbers, phone numbers, and medical data [10]. Further large-scale patient medial information leaks have been reported from Chinese hospitals and universities [10]. So far, the legal repercussions for Chinese entities insufficiently securing private medical data are unknown, though non-medical personal data leaks provide some guidance for future penalties. After China’s Cybersecurity Law came into effect in 2017, significant fines were issued to the Luoyang Beikong Water Group in Henan Province, China, when its data management system was breached. Found responsible for failing to properly secure its data, the company received a fine of RMB 80,000 and three managers took on a collective fine of RMB 35,000 [30].

The healthcare sector in the U.S. perennially experiences the highest costs incurred for data leaks compared to all other commercial sectors, with hospitals experiencing 30 percent of all large data breaches [31]. Within the U.S., the average overall cost to an organization with a medical data leak was 10.1 million USD from March 2021 to March 2022 [32]. These costs include regulatory resolution, victim compensation, lost revenue and information recovery, lost productivity, among others. The average cost per each medical record breached has risen substantially in the U.S. over the last decade, with an average per record cost of 294 USD in 2010, 363 USD in 2015, and 429 USD in 2019 [33]. Notable U.S. and EU medical breaches and corresponding fines include Advocate Health Care Network’s 5.55 million USD settlement with the U.S. Health and Human Services Department (HHS) [34], Excellus Health Plan’s 5.1 million USD settlement with HHS [35], and Dedalus Biologie’s 1.5-million-euro settlement with the French Lead Supervisory Authority (LSA) [36], all for data breaches. Despite the risk of massive financial losses for medical data breaches, only 23 percent of U.S. healthcare organizations employed what are considered complete security automation tools in the year 2020 [37].

### 3.2. Legislation

China’s current legal system mainly includes ten laws, nine administrative regulations, and five national standards for privacy protection in medical AI (see Table 1). Among them, the PIPL, Civil Code, Network Security Law, Data Security Law, and other laws constitute a normative, systematic, and complete personal information protection system, providing basic legal protections for personal information.

### 3.3. Similarities and Differences in Personal Medical Information Protection

There are currently two main international legislative models used for protecting privacy interests in the healthcare industry. One is to separate healthcare information from personal information and protect it through separate legislation. Representing this model is the U.S.’s Health Insurance Portability and Accountability Act of 1996 (HIPAA), the Standards for Privacy of Individually Identifiable Health Information of 2000 (commonly referred to as the Privacy Rule), and the Health Information Technology for Economic and Clinical Health Act (HITECH) of 2009, which provides a systematic and nationwide framework for the protection of private health information. Alternatively, the EU approach protects personal healthcare information together with other personal information in an integrated manner, employing regulations such as the EU Data Protection Directive of 1995 (95/46/EC) and the General Data Protection Regulation of 2018 (GDPR). China’s PIPL attempts to comprehensively protect personal information. In regulating healthcare information, the following differences were found between China, the U.S., and EU (Table 2).

#### 3.3.1. Defining Healthcare Data

China’s PIPL does not define “healthcare information” directly, only stipulating, in Article 28, that medical health information is sensitive personal information and the rules for handling sensitive personal information apply [39]. There are definitions of concepts related to healthcare information in other relevant laws and regulations (see “Medical Health Care Big Data” in the Administrative Measures on National Health and Medical Care Big Data, Standards, Security, and Services (Trial) [11], and “Population Health Information” in the Administration of Population Health Information (Trial) [62]), however, they are not unified. The EU’s GDPR, Article 34, clearly defines “genetic data” and “data concerning health” [63]. The U.S.’s HIPAA Privacy Rule, 45 C.F.R. § 160.103, is more specific in that it protects all “individually identifiable health information” held or transmitted by a covered entity or its business associates, in any form or media, whether electronic, paper, or oral [64]. The Privacy Rule refers to this information as “protected health information (PHI)”, wherein “individually identifiable health information” is information, including demographic data, that relates to: (1) the individual’s past, present, or future health or condition; (2) the provision of health care to the individual; or (3) the payment for the provision of health care to the individual, and that identifies the individual or for which there is a reasonable basis to believe it can be used to identify the individual. Individually identifiable health information includes many common identifiers (e.g., name, address, birth date, and social security number) [64]. 

#### 3.3.2. Consent Model

The PIPL establishes some rules for handling personal information, with “notification and consent” as the core. It does not specify methods to obtain consent for processing personal information. However, according to Article 28 of the PIPL, medical and health information is “sensitive personal information” in China, and “specific purpose”, “sufficient necessity”, and “stringent protective measures” are required for handling sensitive personal information [39]. As such, “separate consent” from individuals, as per Article 29 of the PIPL, must be obtained for the handling of sensitive personal information.

Separate consent is unique to the PIPL. It is a protective measure stating that the processor of personal information should not obtain the consent of the individual in general by mixing different content, purposes of processing, and types of personal information processing activities requiring consent [65]. Instead, there should be separate consent between significantly different personal information collection undertakings. This regulation imposes stricter requirements for handling sensitive personal information. Though, the PIPL does not clarify whether implied consent can be adopted. Whereas in the HIPAA, 45 C.F.R. § 164.508, individual consent must be in writing for the processing of medical information [64]. The GDPR, Article 4, Paragraph 11, provides four elements that must be present for valid consent: (1) freely given; (2) specific; (3) informed; and (4) unambiguous. The GDPR is more open about the format of consent in processing personal data, as it can be in writing or as an oral statement [66]. Article 32 of the GDPR’s introduction states that consent may be given through written statements, this includes electronic methods and ticking boxes when visiting websites, or through oral statements. Electronic informed consent, which uses online forms for consent in health data processing, and dynamic consent, which permits the periodic modification of consent, are not uncommon [1].

#### 3.3.3. Exceptions to Consent and Withdrawing Consent

The PIPL identifies six exceptions to informed consent, which are underwritten by “other circumstances specified by laws and administrative regulations” as a backing clause [39]. This can be applied, to a certain extent, to the healthcare industry. However, compared to specific consent exception scenarios in the EU and U.S. models, particularly the HIPAA Act [64], which establishes rules for data processing in specific scenarios and under different exceptions, the operability of Chinese legislation is clearly inadequate. 

In the HIPAA Act, 45 C.F.R. § 164.502(a)(1), exceptions for authorization are provided to balance the interests of the public and personal privacy, including when information is used in medical research and for other public health or healthcare activities [64]. For instance, entities may use or disclose protected health information for research purposes with waivers from ethics or privacy committees. Privacy committees must be composed of members with diverse backgrounds and adequate competencies, including at least one member who is unrelated to the protected entities, research sponsors, or investigators. The waiver criteria involves the following: (1) the use or disclosure involves no more than minimal risk; (2) the research could not practicably be conducted without the exemption; (3) the privacy risk is reasonable in relation to anticipated benefits; (4) a plan for the destruction of identifiers is required unless there is a health or research justification for retaining them; and (5) there is written assurance that the data will not be reused or disclosed to others, except for research oversight or additional research that would also qualify for a waiver [67].

The GDPR, Article 9, establishes exceptions for processing special categories of personal data [63]. For health data, personal data processing is prohibited unless certain conditions are met. For example, regarding public health, data processing is necessary to protect against serious cross-border threats to public health or to ensure high standards of quality and safety for healthcare (this largely involves medicinal products and medical devices). 

In contrast, PIPL exceptions for healthcare are formulated as “necessary to respond to sudden public health incidents or protect the life, health, and the security of property of natural persons under emergency conditions” [39]. By adopting “sudden” and “emergency” as the extent of the exceptions, it indicates that the scope of the exceptions is narrower compared to “prevention” in the GDPR. Uncertainty and discretion have led to the increased use of the safer approach to processing personal data: directly obtaining user consent [68]. However, requiring the consent of each individual patient before the totality of the data can be used may disrupt large-scale research projects. 

Regarding withdrawing consent, Article 15 of the PIPL stipulates the individual’s right to withdraw consent, where “personal information processing is based on individual consent, an individual shall have the right to withdraw consent. Personal information processors shall provide convenient means for an individual to withdraw consent”. [39] The HIPAA, 45 C.F.R. 46.116(a)(8), provides that an individual generally has the right to revoke any authorization that has been granted, provided the revocation is in writing [64]. GDPR withdrawal regulations, stipulated in Article 7, Paragraph 3, require that data subjects be provided with the opportunity to withdraw consent as easily as it was to give the consent [63].

#### 3.3.4. Right to Erasure and Right to Be Forgotten

Article 47 of the PIPL conveys the right to erase personal information based on provisions in the Civil Code. The information processor is obliged to delete information when one of the following circumstances are met: (1) the purpose of processing has been achieved or cannot be achieved, or such information is no longer necessary for achieving the purpose of processing; (2) the personal information processor ceases to provide products or services, or the storage period has expired; (3) the individual withdraws consent; and (4) the personal information processor has processed personal information in violation of laws, administrative regulations, or the agreement of the parties [39]. 

The GDPR, Article 17, also establishes the right to be forgotten. When data controllers make personal data publicly available, they are obliged, pursuant to a subject’s request, to erase the subject’s personal data. The data controller, considering the available technology and the cost of implementation, shall take reasonable steps, including technical measures, to inform other controllers processing the relevant personal data that the data subject has requested erasure by the controllers of any electronic links, copies, or replications of their personal data. Hence, controllers do not simply delete the data under their control but are also obliged to notify other third parties to cease using and delete the data that they have publicly disseminated. There is no similar provision in the U.S. Privacy Rule. This Rule requires no modification of data for federal and state medical records or other records. For example, 45 C.F.R. § 164.530(j) requires that its privacy policies and procedures, notices of privacy practices, complaint handling, and other actions be documented and retained for at least six years after the date of creation or its last effective date [64]. Federal health laws, 45 C.F.R. § 164.316, require the retention of medical records, billing records, compliance records, and other records for at least six years [64]. There are important clinical reasons for this record retention requirement. For example, clinicians need to know if patients are allergic to different drugs or have had adverse drug reactions in the past. Old medical records are critical in providing this information and preventing adverse drug reactions and other harm [66].

#### 3.3.5. Data Protection Officer System

Currently, China does not employ a special data protection officer system, and its relevant laws and regulations on network security are weak. For example, China’s “Cyber Security Law”, Article 40, requires that network operators manage network security [40], and the PIPL, Article 9, requires that entities establish a person to manage personal information protection [39]. However, the regulations for data protection officers are unclear, and the regulations for enterprises to establish data protection officers are relatively vague. Penalties for noncompliance are also relatively light. 

According to the GDPR, Article 37, data controllers or processors whose core activities include processing large amounts of personal data (including health-related data) should appoint a data protection officer [63]. Enterprises failing to establish a data protection officer, per Article 83, Paragraph 4, are subject to an administrative penalty of up to 10 million euros or up to 2 percent of the enterprises total worldwide annual turnover of the preceding fiscal year, whichever is higher [63]. The HIPAA, 45 C.F.R. § 164.530(a), requires covered entities to establish a privacy officer responsible for developing and implementing privacy policies, and to establish a contact person or liaison office responsible for receiving complaints and providing affected individuals or concerned party’s information about privacy incidents [64].

In summary, it is evident that although Chinese legislators are aware of the special protection needs required of medical and health information, their regulatory path is more abstract and generalized compared to the U.S. and EU. The Chinese protection of medical and health information applies mainly to rules of consent and authorization for sensitive personal information and other stricter rules for personal information processing. Protections for the general processing of medical and health information in AI scenarios is rather weak.

## 4. Discussion

### 4.1. Conceptual Distinctions between Personal Information and Privacy

The first issue requiring consideration is the conceptualization of privacy and the definitional interrelationship between privacy and personal information. Determining the concept and scope of privacy, which is a premise for resolving legal disputes, maintains certain dilemmas. Information technology has rendered traditional private sector privacy protection boundaries somewhat ambiguous, as personal information often flows freely between the private and public sectors. The public’s reliance on smart technology has also precipitated an increased integration of private information and cyberspace, reducing privacy expectations, normalizing personal information sharing, and creating a massive online market where distributing personal information of all kinds is profitable [69].

From a legal perspective, privacy and personal information are closely related, though, not completely identical. The two are intertwined, overlapping and distinguishable. Personal information includes both private information, such as private conversations that can be digitized through technical processing—this may be categorized as personal information because of its identifiability. At the same time, it also includes non-private information, such as the disclosure of an individual’s telephone number without his or her consent. This is personal information that is no longer relevant to personal privacy.

The intersectionality of privacy and personal information has led to differences in the choice of legal protection models for privacy and personal information rights in various countries. The U.S. adopts the model of protecting personal information in a unified manner, embracing personal information protections through the concept of “information privacy”. For example, the U.S. Privacy Act of 1974 is based on both the protection of privacy and the protection of personal information through the right to privacy [70]. In the EU, the protection of personal data has been gradually separated from the protection of privacy. For example, the EU Data Protection Directive (95/46/EC) of 1995 established “privacy” as one of the values of personal data protection [71]. However, the GDPR replaced “the right to privacy in the processing of personal data” in Article 1 of the Data Protection Directive with “the right to the protection of personal data” [63], highlighting the independent nature of personal data protection.

Chinese law adopts a separate protection model for the rights of privacy and personal information. Article 1034, Paragraph 3, of the Civil Code establishes that private information in personal information shall be given priority within provisions on personal information protection. As a result, it is difficult to establish standards for testing the privacy of personal information in judicial practice. In addition to the classification of private information and non-private information, the PIPL adds another classification standard for personal information: sensitive personal information and general personal information. Article 28 of the PIPL stipulates that “sensitive personal information” is personal information that, once leaked or used illegally, may easily lead to infringements on the personal dignity of natural persons or may endanger personal safety or property [39]. Sensitive personal information includes biometrics, religious beliefs, specific identities, medical health status, financial accounts, people’s locations, and the personal information of minors under the age of 14 years. This has resulted in a new problem: how to define the relationship between sensitive personal information and private personal information, and whether the two are equivalent. This issue has become controversial among academics. According to some, sensitive personal information refers to personal information that is highly private and the disclosure or use of which will have a significant impact on an individual [72].

### 4.2. Collecting and Using Data

Privacy leaks generally occur in one of three AI-based data processing stages: first, the data collection stage, where an information service platform may collect personal information beyond what is necessary or collect sensitive personal information without subjects’ knowledge, causing personal privacy infringement. Second, the data use stage, where improperly using data after data collection or sharing users’ personal information between different platforms without informing users produces privacy leaks. Third, the algorithmic prediction stage, where advanced computer programs and AI-led big data technologies analyze people’s personal information for hidden information. What matters may no longer be the data itself, but rather the additional, often hidden, information obtained using AI algorithms. Algorithm technology and its prediction accuracy have reached levels of precision that often eliminate the need for direct information. This creates a risk for privacy leaks from algorithmic predictions, which are most likely to happen under auxiliary diagnosis and treatment scenarios. Thus, the results predicted by deep learning algorithms are likely an extension of private information and should be protected by law.

At present, the rules for handling personal information established by the PIPL mainly include advanced notice, obtaining personal consent, no excessive collection, and handling (including collecting) sensitive personal information only when specific purposes, sufficient necessity, and strict protection measures are adopted. However, with the development of AI technology, privacy risks are increasing, and informed consent rules and the de-identification of personal information still face challenges.

#### 4.2.1. Challenges to Informed Consent Rules

Privacy protection requires self-determination over privacy practices, wherein individuals independently decide when, how, and to what extent their personal information is communicated to others. The informed consent rules in the PIPL are based on the protection concept of ensuring that information subjects have control over their personal information; these concepts are also emphasized in U.S. and EU law. However, the informed consent model is weakened within the context of big data, AI, and healthcare, as it is often difficult to be clearly informed of the purpose and use of personal information.

The sources of healthcare data are becoming more diverse and abundant, including information recorded in wearable devices, genetic information from genome sequencing, digital and electronic medical records, radiological images, and data collected from hospital wards. Data collectors are no longer limited to medical institutions. Data can come from pharmaceutical companies, medical device manufacturers, health service companies, and others. The relationship between individuals and data collectors no longer follows the traditional single-line connection between patients and their immediate medical institutions. Instead, this connection has expanded into an association of multiple entities, such as medical data intermediaries, medical application service providers, and subsequent users of medical data. The scale and complexity of big data in healthcare makes it difficult for individuals to fully track all the destinations and potential uses of their healthcare information.

AI technologies often process millions of datapoints to make predictions, regularly employing algorithms and decision-making methods that are difficult for computer programmers, data specialists, and information subjects to decipher. This is known as the “black-box effect”, and in the medical field it poses new challenges to privacy protection. Although China’s Civil Code and the PIPL stipulate that the purpose, manner, and scope of processing personal information should be made clear, the provision is vague and does not specify what processing rules and content should be disclosed, whether it is the code, the underlying data, or the decision logic of the algorithm. The GDPR has taken the lead in establishing algorithmic rules for companies and other entities [73]. These rules increase algorithmic transparency, thus reducing privacy risks associated with algorithmic ambiguity. However, there is still controversy in the academic community as to whether algorithmic decision-making is interpretable, and whether the right of algorithmic interpretation should be established.

In addition, the application of AI in the healthcare industry, especially the use of deep learning technologies to assist in diagnosis or predictive diagnosis, requires large amounts of data input. Ensuring individual consent for all data in these massive inputs is often cumbersome, and, at times, unrealistic. The development of technology and innovation in the medical field will be hindered if each personal information processing event requires known and documented individual consent from all patients or involved parties.

#### 4.2.2. De-identification Protections May Not Protect Personal Privacy

The PIPL only protects personal information that is identifiable, meaning that individual consent is not required to process information unconnected to specific individuals after anonymization. Therefore, there is a great deal of data which requires anonymization before processing. However, in the field of healthcare, some medical and health information cannot be anonymized, otherwise it will lose its original value and meaning. Individual patients are specifically targeted in medical diagnosis services, wherein the recording and analysis of health information provides medical diagnosis results. When this information is anonymized, immediate assistance might not be available when the health and life of information subjects is under threat. For example, in the mid-1990s, the U.S. Congress criticized a study employing anonymization conducted by the Centers for Disease Control and Prevention on the prevalence of HIV among newborns. Anonymous record keeping prevented health authorities from returning test results to providers of care for HIV-positive infants, and, as a result, these children could not receive immediate treatment [67]. Stripping too much relevant information to protect privacy also has the potential to reduce the scientific and research value of the data.

The de-identification of personal information is important when using healthcare information for subject research, drug development, and other fields. Generally, hospitals take certain security measures when applying patient medical and health information to research applications. This includes having subjects sign confidentiality agreements, data desensitization, anonymization techniques, and the timely destruction of data. However, with technological developments, completely unidentifiable information may no longer exist. The development of reverse identification technology has increased the risk of data re-identification [74]. The high-risk nature of identifiability in cyberspace also makes it difficult to achieve complete “anonymity” on the internet, as almost all network data can be linked to some identifiable person. Anonymized information can be combined with other sources of personal information to piece together information about individuals addresses, socioeconomic backgrounds, or even comprehensive identity information. In summary, the distinction between personal and non-personal information is dynamic and the boundaries between the two often depend on rapidly expanding technological developments, making it difficult to use “identifiability” as the standard for personal information protection.

### 4.3. Cross-Border Data Flow

The availability and free flow of data are often considered important factors in the development of AI technology. However, cross-border data flow and privacy protections may conflict. Different countries have different privacy and security standards and adopt different models for cross-border data flow. For China, data protection is closely related to national security and involves the maintenance of national sovereignty and public security. Therefore, China has always been cautious about cross-border data flow and prefers protecting personal data and the national jurisdiction at the expense of certain freedoms in cross-border information transfer. In terms of national law, Chapter 3 of the PIPL establishes strict review requirements for cross-border transfers of personal information. Article 38 requires that the cross-border flow of personal information be subject to security assessments organized by the National Cyberspace Department, certified by a relevant specialized institution for personal information protection, or concluded in contracts with overseas recipients in accordance with standard contracts formulated by the National Cyberspace Department (specifying the rights and obligations of all parties) [75]. Article 39 requires separate consent from all subjects involved [75], and Article 55 requires an impact assessment conducted in advance for personal information protection [75]. In the realm of international law, when China signs free trade agreements with other countries, most of these agreements do not include provisions on cross-border data flow. China signed two free trade agreements containing provisions on cross-border data flow in 2015, with both South Korea and Australia [76,77]. China and Chile also included separate chapters on e-commerce when amending their free trade agreement in 2019 [78]. However, these chapters only addressed issues within the scope of e-commerce, such as a moratorium on electronic tariffs, electronic authentication, electronic signatures, protection of personal information in e-commerce, and paperless transactions, without addressing internet and data regulation issues.

In the EU, the right to privacy and the protection of personal data are fundamental and binding rights, hence, the GDPR has imposed strict conditions for cross-border data flow, restricting personal data transfers outside the European Economic Area (EEA) and disallowing data transfers to countries not providing adequate protection. The U.S., on the other hand, has opted for a more liberal regulatory model for cross-border data flow, weakening protections for personal data. However, at the same time, the U.S. is leveraging its technological and commercial advantages to expand its jurisdiction over personal information [79]. The U.S. includes binding provisions in most of its leading regional agreements, such as the USMCA and the U.S.-Japan Digital Trade Agreement, requiring parties to allow (or not restrict) the transfer of information across borders by electronic means [80].

In the context of rapid developments in big data, the cross-border flow of personal data is an important channel for the development of medical AI, but it may also infringe on the privacy rights of information subjects. The stringent conditions of the GDPR provides strong institutional support for personal privacy protection, however, it also increases compliance costs for the exportation of data, hinders cross-border data flow, and is detrimental to AI development. The U.S. advocates free data flow in pursuit of maximizing commercial interests, at the cost of greatly increasing the risk for privacy leaks. When constructing a reasonable model of cross-border data flow, it is necessary to consider both economic and privacy concerns, balancing technological and economic development with privacy protections.

## 5. Conclusions

While AI technology broadens pathways for healthcare development, there are serious risks and considerations associated with patient privacy that need to be addressed, specifically the need for reasonable regulation in handling patient healthcare information before, during, and after it is used for AI applications. The construction of a legal protection system must strike a balance between privacy protection and technological innovation. When implementing legal regulations, it is necessary to prevent healthcare providers from infringing on privacy rights in pursuit of boundless technological development to occupy the market and satisfy stakeholders. Technological innovation and company profits should not be at the expense of privacy rights, as the goal of any technological development is to serve human beings.

At the same time, the excessive protection of privacy hinders technological development, as the development of AI technology relies on large amounts of data as the basis for algorithmic learning. Eliminating the use of patient provided data is tantamount to simply giving up on medical AI development. It may be difficult for dispersed individuals to take unified and effective medical countermeasures when dealing with health threats, especially when treating difficult diseases, requiring large amounts of healthcare information for research support. The public, therefore, is dependent upon the innovation and success of healthcare providers. It is necessary to avoid unreasonably harsh privacy requirements in data processing, which might hinder medical AI development. As such, the interests of individuals, and their personal privacy, should be considered alongside the practical and economic interests of healthcare providers. Improving patient privacy protections are conducive to enhancing acceptance and trust in medical AI.

In constructing a legal framework for privacy protection, China should consider promulgating a special law in concert with the PIPL, and further a dual regulatory structure of “basic law + special law” to refine medical and health data processing rules. Within this framework, the following actions should be taken: (1) Unify concepts related to healthcare data in various legal documents. Due to the current lack of uniform legislation on healthcare data in China, the existing legal documents, which have similar legal names and definitions for healthcare data, have created difficulties in regulating the collection, storage, use, and transmission of medical information. (2) Classify data and formulate different rules for special types of data. Chinese law is unclear concerning which types of data qualify for special protections, and how this special data should be protected. The penalties for a failure to protect special data should also be detailed. It is recommended that Chinese legislators devise clear categories for special types of data. The type of data falling into each category should be clearly defined, as should the rules for processing each category. (3) The interactive nature of consent should be enhanced, through electronic informed consent and dynamic consent, to make “informed consent” more effective. (4) Construct a strict accountability mechanism to ensure that privacy rights violations in medical AI can be regulated and corrected. This should include prompt and strict penalties for governments and companies inappropriately deploying AI technologies in healthcare. When AI algorithms perform machine learning and data mining resulting in breaches of sensitive personal information and infringements of individual privacy rights, all parties in the data supply chain, from top to bottom, should be held legally responsible unless information is provided proving they were not at fault.

It is crucial that medical institutions and related data processors implement information security and strengthen the internal control and management of sensitive personal information in accordance with legal provisions. EU privacy protection management, as it operates through the data protection officer system, should become a significant reference for China. China should legislate that organizations dealing with healthcare sensitive information on a large scale must establish special data protection officers (DPOs), strictly stipulate the appointment conditions of DPOs (officers must have professional expertise in data protection laws and practices), clarify the responsibilities of DPOs, and impose severe penalties against DPOs violating regulations.

Further, to aid in developing an international network for data governance in healthcare and open channels for information sharing, China should actively promote and participate in building an international legislative framework for data governance in healthcare. Considering the differences and conflicts in data governance rules among countries, it would be easier to strengthen data protection and promote cross-border data flow through international soft law. As a member of the Asia-Pacific Economic Cooperation (APEC), China should take advantage of APEC’s relatively mature privacy framework to use as reference for an international data governance network for healthcare, and should also consider WHO guidance on ethics and governance of AI in healthcare to facilitate cross-border data flow.

## Figures and Tables

**Table 1:** Chinese laws, regulations, and national standards for privacy protection in medical AI.

Type of Regulation	Title of Regulation	Date Effective	Relevant Clauses
**Laws**	Civil Code [38]	1 January 2021	Chapter 6, Articles 1032 and 1039
	Personal Information Protection Act [39]	1 November 2021	Chapter 2, Articles 28 and 32
	Cybersecurity Law [40]	1 June 2017	Articles 41 and 42
	Data Security Law [41]	1 September 2021	Article 32
	Promotion of Basic Medical and Health Care Law [42]	1 June 2020	Article 33
	Mental Health Law (2018 Amendment) [43]	27 April 2018	Article 4
	Prevention and Treatment of Infectious Diseases Law (2013 Amendment) [44]	29 June 2013	Article 12
	Law on Doctors [45]	1 March 2022	Article 23
	Maternal and Infant Health Care (2017 Amendment) [46]	5 November 2017	Article 34
	Criminal Law (2020 Amendment) [47]	1 March 2021	Article 253
**Regulations**	Regulation on the Prevention and Treatment of HIV/AIDS (2019 Revision) [48]	2 March 2019	Article 39
	Nurses Regulation (2020 Revision) [49]	27 March 2020	Article 18
	Provisions on the Cyber Protection of Children’s Personal Information [50]	1 October 2019	Article 7
	Regulations on Medical Record Management in Medical Institutions (Version 2013) [51]	1 January 2014	Article 6
	Measures for the Administration of Population Health Information (for Trial Implementation) [52]	5 May 2014	Articles 2, 3, and 6
	Management Standards for the Application of Electronic Medical Records (for Trial Implementation) [53]	1 April 2017	Article 8
	Measures for the Administration of National Health and Medical Big Data Standards, Security and Services (for Trial Implementation) [54]	13 July 2018	Article 2
	Measures for the Administration of Internet Diagnosis and Treatment (for Trial Implementation) [55]	17 July 2018	Article 20
	Measures for the Administration of Internet Hospitals (for Trial Implementation) [56]	17 July 2018	Article 23
**National Standards**	Health Informatics—Guidelines on Data Protection to Facilitate Trans-border Flows of Personal Health Information [57]	1 May 2011	It provides general requirements for protecting personal health data transmitted across borders.
	Information Security Technology -Personal Information Security Specification [58]	1 October 2020	It addresses security problems influencing personal information and regulates the behavior of personal information controllers in the collection, storage, use, sharing, transfer, public disclosure, and other information processing in accordance with the “Cybersecurity Law of the People’s Republic of China” and other relevant laws. It aims to restrain the illegal collection, misuse, and leakage of personal information, maximizing protections for the legitimate rights and interests of individuals and the public.
	Information Security Technology–Guidance for Personal Information Security Impact Assessments [59]	1 June 2021	It specifies the basic principles for impact assessments and the implementation of personal information security.
	Information Security Technology–Guide for De-identifying Personal Information [60]	1 March 2020	It aims to protect personal information, while, at the same time, promotes sharing and using data. Guidelines and standards for the de-identification of personal information are formulated.
	Information Security Technology–Guide for Health Data Security [61]	1 July 2021	It provides that healthcare data security is related to patient safety, personal information security, the public interest, and national security. As such, to better protect healthcare data and regulate and promote healthcare data sharing, particularly the open application of healthcare data and the development of the healthcare industry, healthcare data security guidelines are formulated.

**Table 2:** Differences between China, the U.S., and EU in regulating healthcare information.

	China	United States	European Union
**Definition**	“Sensitive personal information” includes medical health information. (PIPL, Article 28, Paragraph 1) “Health and medical big data standards” refer to healthcare data generated in the process of disease prevention and health management. (Measures for Administration of National Health and Medical Big Data Standards, Security and Services, Article 4) “Population health information” refers to basic population information, medical and health service information, et cetera, generated in the process of service and management by various types of medical, health, and family planning service institutions at all levels in accordance with national laws, regulations, and job responsibilities. (Measures for the Administration of Population Health Information, Article 3)	“Individually identifiable health information” is information, including demographic data that relates to: (1) the individual’s past, present, or future health or condition; (2) the provision of health care to the individual; or (3) the payment for the provision of health care to the individual, and that identifies the individual or for which there is a reasonable basis to believe it can be used to identify the individual. Individually identifiable health information includes many common identifiers (e.g., name, address, birth date, and social security number). (45 C.F.R. § 160.103)	“Genetic data” means personal data relating to the inherited or acquired genetic characteristics of a natural person which give unique information about the physiology or the health of that natural person and which result from an analysis of a biological sample from the natural person in question. “Data concerning health” refers to personal data related to the physical or mental health of a natural person, including the provision of health care services, which reveal information about his or her health status. (GDPR, Article 4)
**Consent Model**	For the processing of sensitive personal information, individual and separate consent shall be obtained. Where other laws or administrative regulations provide that written consent shall be obtained for the processing of sensitive personal information, such provisions shall prevail. (PIPL, Article 29)	Individual authorization consent for medical health information needs to be in writing. (45 C.F.R. § 164.508)	Consent can be in writing (including electronically) or in the form of an oral statement. (GDPR introduction, Article 32)
**Exceptions to Consent**	The processor of personal information does not require the consent of an individual to process their personal information if one of the following circumstances exists: … (3) in response to a public health emergency, or necessary to protect the life, health, and property of natural persons in an emergency. (PIPL, Article 13)	Provides exceptions from the requirement for authorization for— (1) individuals (unless access or accounting disclosure requirement); (2) treatment, payment, and health care operations; (3) opportunities for consent or objection; (4) other permissible use and disclosure events; (5) public interest and welfare activities; and (6) limited data sets used in research, public health, or health care operations. (45 C.F.R. § 164.502(a)(1))	Exceptions that do not require the explicit consent of the data subject are for preventive or clinical medical purposes, or for the assessment of an employee’s work capacity, or in the public health field for the achievement of public interest. (GDPR, Article 9, Paragraph 2)
**Withdrawal of Consent**	Where personal information processing is based on individual consent, an individual shall have the right to withdraw consent. Personal information processors shall provide convenient ways for individuals to withdraw consent. (PIPL, Article 15)	An individual generally has the right to revoke a granted authorization in any implementation, so long as the revocation is in writing. (45 C.F.R. § 164.508(b)(5))	It should be as easy for the data subject to withdraw his consent as it is for the data subject to express it. (GDPR, Article 7, Paragraph 3)
**Right to Erasure and Right to be Forgotten**	Right to erasure: in any of the following circumstances, a personal information processor shall take the initiative to erase personal information, and an individual has the right to request the deletion of personal information if the personal information processor fails to erase the information: (1) the purpose of processing has been achieved, cannot be achieved, or it is no longer necessary to achieve the purpose of processing; (2) the processor of personal information ceases to provide products or services, or when the storage period has expired; (3) the individual withdraws consent; (4) when the processor of personal information processes personal information in violation of laws, administrative regulations, or agreements; (5) other circumstances as provided by laws and administrative regulations. (PIPL, Article 47)	Must maintain its privacy policies and procedures, notices of privacy practices, complaint handling, and other actions, activities, and designations that the Privacy Rule requires to be documented must be retained for at least six years after the date of creation or its last effective date (45 C.F.R. § 164.530(j))	Right to erasure (‘right to be forgotten’): when a data controller has made the personal data publicly available and is obliged to erase the personal data, the data controller shall take reasonable steps, including technical measures, to inform controllers who are processing the personal data that the data subject has requested the erasure by such controllers of any links to, or copy or replication of those personal data. (GDPR, Article 17, Paragraph 2)
**Data Protection Officer System**	The network operator shall determine the person in charge of network security in accordance with the requirements of the network security protection system. (Cybersecurity Law, Article 21) Where the quantity of personal information processed by a processor reaches that specified by the State Cyberspace Administration, the processor shall designate a person in charge of personal information protection to be responsible for supervising the processing of personal information and the adopted protection measures. (PIPL, Article 52)	Covered entities must designate a privacy officer responsible for developing and implementing their privacy policies and a contact person or liaison office responsible for receiving complaints and providing individuals with information about privacy incidents of the covered entity. (45 C.F.R. § 164.530(a))	A data protection officer shall be appointed if the core activities of the controller or processor of the data include the processing of large-scale special types of personal data (which includes data related to the health of natural persons). (GDPR, Article 37)

## Data Availability

The data presented in this study are available on request from the authors.
